# TLR-2 Recognizes *Propionibacterium acnes* CAMP Factor 1 from Highly Inflammatory Strains

**DOI:** 10.1371/journal.pone.0167237

**Published:** 2016-11-30

**Authors:** Coralie Lheure, Philippe Alain Grange, Guillaume Ollagnier, Philippe Morand, Nathalie Désiré, Sophie Sayon, Stéphane Corvec, Jöel Raingeaud, Anne-Geneviève Marcelin, Vincent Calvez, Amir Khammari, Frédéric Batteux, Brigitte Dréno, Nicolas Dupin

**Affiliations:** 1 Université Sorbonne Paris Descartes, Faculté de Médecine, INSERM, Institut Cochin, Laboratoire de Dermatologie-CNR Syphilis, Paris, France; 2 AP-HP, Groupe Hospitalier Paris Centre Cochin-Hôtel Dieu-Broca, Service de Bactériologie-CNR Streptocoques, Paris, France; 3 Sorbonne Université, UPMC Université Paris 06, INSERM, Institut Pierre Louis d’Epidémiologie et de Santé Publique, Paris, France; 4 AP-HP, Groupe hospitalier Pitié Salpêtrière, Laboratoire de Virologie, Paris, France; 5 Service de Bactériologie-Hygiène hospitalière, CHU de Nantes, Nantes, France; 6 UMR8126, Institut Gustave Roussy, Villejuif, France; 7 CHU, service de dermatologie, CIC, Hôtel Dieu, Nantes, Hôtel Dieu, Nantes, France; 8 AP-HP, Groupe Hospitalier Paris Centre Cochin-Hôtel Dieu-Broca, Service d’Immunologie Biologique, Paris, France; 9 AP-HP, Groupe Hospitalier Paris Centre Cochin-Hôtel Dieu-Broca, Service de Dermatologie-Vénéréologie, Paris, France; Aarhus Universitet, DENMARK

## Abstract

**Background:**

*Propionibacterium acnes* (*P*. *acnes*) is an anaerobic, Gram-positive bacteria encountered in inflammatory acne lesions, particularly in the pilosebaceous follicle. *P*. *acnes* triggers a strong immune response involving keratinocytes, sebocytes and monocytes, the target cells during acne development. Lipoteicoic acid and peptidoglycan induce the inflammatory reaction, but no *P*. *acnes* surface protein interacting with Toll-like receptors has been identified. *P*. *acnes* surface proteins have been extracted by lithium stripping and shown to induce CXCL8 production by keratinocytes.

**Methodology and principal findings:**

Far-western blotting identified two surface proteins, of 24.5- and 27.5-kDa in size, specifically recognized by TLR2. These proteins were characterized, by LC-MS/MS, as CAMP factor 1 devoid of its signal peptide sequence, as shown by N-terminal sequencing. Purified CAMP factor 1 induces CXCL8 production by activating the CXCL8 gene promoter, triggering the synthesis of CXCL8 mRNA. Antibodies against TLR2 significantly decreased the CXCL8 response. For the 27 *P*. *acnes* strains used in this study, CAMP1-TLR2 binding intensity was modulated and appeared to be strong in type IB and II strains, which produced large amounts of CXCL8, whereas most of the type IA_1_ and IA_2_ strains presented little or no CAMP1-TLR2 binding and low levels of CXCL8 production. The nucleotide sequence of CAMP factor displays a major polymorphism, defining two distinct genetic groups corresponding to CAMP factor 1 with 14 amino-acid changes from strains phylotyped II with moderate and high levels of CAMP1-TLR2 binding activity, and CAMP factor 1 containing 0, 1 or 2 amino-acid changes from strains phylotyped IA_1_, IA_2_, or IB presenting no, weak or moderate CAMP1-TLR2 binding.

**Conclusions:**

Our findings indicate that CAMP factor 1 may contribute to *P*. *acnes* virulence, by amplifying the inflammation reaction through direct interaction with TLR2.

## Introduction

*Propionibacterium acnes* (*P*. *acnes*) is an anaerobic Gram-positive bacterium frequently present in the normal human skin microbiota, where it accumulates preferentially in the pilosebaceaous units in individuals with and without acne [1]. This bacterium has long been considered to be commensal, but there is growing evidence that it also acts as an opportunistic pathogen, causing infections associated with diverse implants, including breast implants, neurosurgical shunts, cardiovascular devices, ocular implants and prosthetic joints, and that specific clones of *P*. *acnes* are associated with acne [2, 3, 4, 5, 6, 7]. *P*. *acnes* is, indeed, best known for its association with acne, a common inflammatory disorder of the sebaceous follicles affecting more than 85% of adolescents but also persisting or occurring in some adults [8]. Acne is a multifactorial disease characterized by an increase in sebum secretion associated with changes in sebum composition induced by androgens, hyperkeratinization leading to the obstruction of sebaceous follicles, changes in *P*. *acnes* protein production and an intense inflammatory reaction, but the exact sequence of these events remains unclear [9, 10, 11]. Studies involving MLST approaches have classified *P*. *acnes* strains into six phylotypes (IA_1_, IA_2_, IB, IC, II and III) according to their ability to induce the production of proinflammatory molecules [12], their association with infections, their biochemical and morphological characteristics and their ability to aggregate [13, 14, 15, 16, 17, 18]. A variable number of tandem repeats-based method was recently developed, to improve genotyping and discriminate between *P*. *acnes* strains [19]. The core genes of *P*. *acnes* seem to be highly conserved between strains, but several non-core loci have been identified that interfere with expression levels and are correlated with the different phylotypes [20]. Indeed, differences have been observed in CXCL8 production by keratinocytes stimulated with different *P*. *acnes* strains [21], together with differences in protein secretion [22]. The IA_1_ phylotype has also been shown to be strongly associated with acne lesions, whereas the type III phylotype is rarely found in these lesions but accounts for 20% of isolates from normal skin. Types IB and II are overrepresented in soft-tissue and implant–associated infections, and in bacteremia [16, 23].

The innate immune response is the body’s first line of defense against infectious agents, and its success is reflected in health and well-being. Pathogen recognition by the innate immune system relies on a limited number of pattern recognition receptors (PRR) that recognize conserved products of microbial metabolism produced by microbial pathogens and known as pathogen-associated molecular patterns (PAMPs). The best-known PRRs are the Toll-like receptors (TLRs). Ten TLRs have been described in mammals and have been classified into two groups: TLRs 1, 2, 4, 5, and 6, localized on the cellular membrane, are activated by extracellular PAMPS; and TLRs 3, 7, 8, 9, localized on intracellular organelles, such as lysosomes and endosomes. Together with TLR1, TLR6 and CD36, TLR2 plays a crucial role in the recognition of peptidoglycan (PGN, a molecule expressed by many bacterial species), lipoproteins, and lipoteichoic acid (LTA) from Gram-positive bacteria, and of lipoarabinomannan from mycobacteria and zymosan from fungal. TLR4 acts together with CD14 and MD2 in the recognition of LPS from Gram-negative bacteria [24].

*P*. *acnes* contributes to the inflammatory lesions of acne by activating innate immunity via the TLR2 expressed on cutaneous cells [25]. *In vitro*, *P*. *acnes* stimulates keratinocytes and monocytes, leading to the production of proinflammatory cytokines (IL-1 α/β, CXCL8, IL-12, TNFα) via TLR2 activation [26, 27, 28], and the generation of reactive oxygen species through the activation of scavenger receptor CD36 [29]. *In vivo*, the genes encoding CXCL8, TLR2, and β-defensin-4 have been shown to be upregulated in acne lesions, together with NF-κB and AP-1, suggesting an activation of TLR2 by *P*. *acnes* [30, 31, 32, 33, 34].

The surface proteins of *P*. *acnes* involved in the activation of TLR2 on keratinocytes and monocytes and leading to inflammatory lesions in acne have yet to be identified. The aim of this study was to identify surface proteins of *P*. *acnes* recognized by TLR2 in several strains with different inflammatory profiles.

## Results

### Identification of *P*. *acnes* surface proteins recognized by TLR2

**Fig 1:**
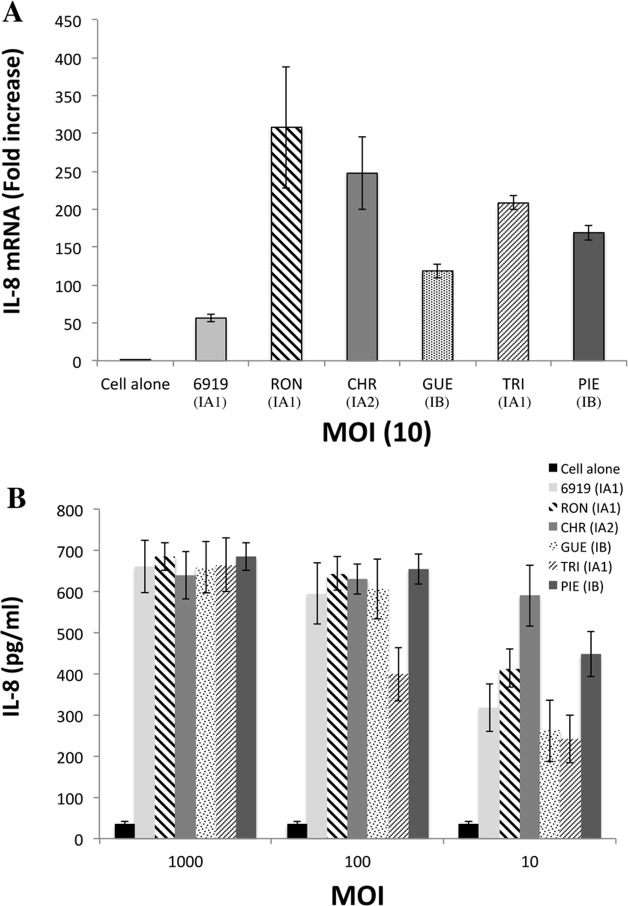
Induction of CXCL8 production in kerationocytes stimulated with whole *P*. *acnes* bacteria. HaCaT cells were incubated for 18 h with whole *P*. *acnes* bacteria (6919, RON, CHR, GUE, TRI, PIE, Table 1) at a MOI of 10 (A) and at MOI of 10, 100, 1000 (B). Total RNA was extracted and CXCL8 mRNA levels were determined by real-time RT-PCR and compared those of GAPDH mRNA (used as the control). The difference is expressed as a fold-change. CXCL8 production was analyzed by ELISA on culture supernatants. Control experiments were run with unstimulated cells. The data are presented as the mean ± standard deviation of three independent experiments. Statistical significance is indicated by * *P* ≤ 0.05, ** *P* ≤ 0.01, *** *P* ≤ 0.001, **** *P* ≤ 0.0001.

**Fig 2:**
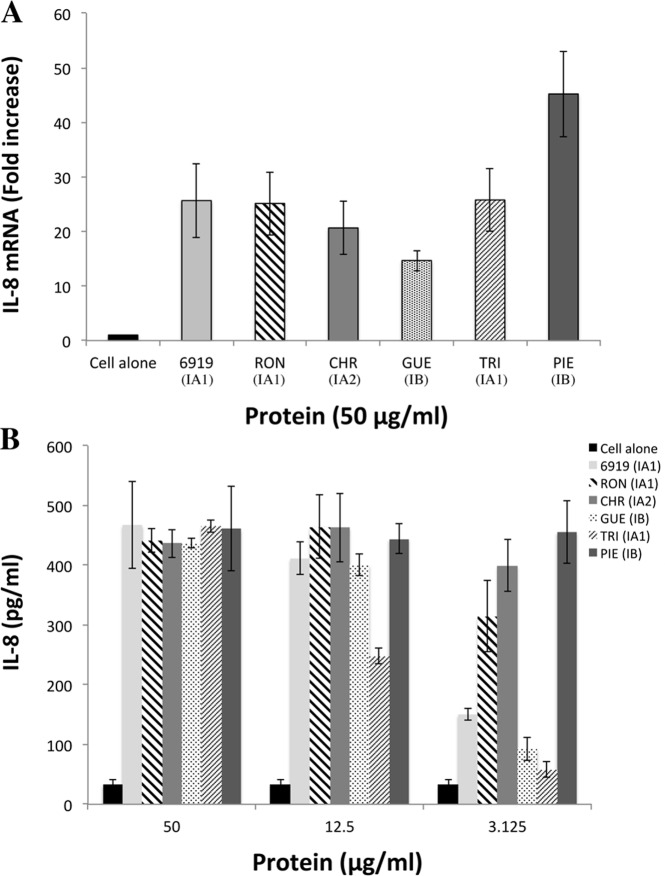
Induction of CXCL8 production in keratinocytes by *P*. *acnes* surface proteins. HaCaT cells were incubated for 18 h with lithium extracts of bacterial proteins at concentrations of 50 μg/ml (A) and 3.12, 12.5, and 50 μg/ml (B). Total RNA was extracted and CXCL8 mRNA levels were determined by real-time RT-PCR and compared with GAPDH mRNA levels (used as the control), with expression as a fold-change. CXCL8 production was assessed by ELISA on culture supernatants. Control experiments were performed with unstimulated cells. Data are presented as the mean ± standard deviation of three independent experiments. Statistical significance is indicated by * *P* ≤ 0.05, ** *P* ≤ 0.01, *** *P* ≤ 0.001, **** *P* ≤ 0.0001.

**Fig 3:**
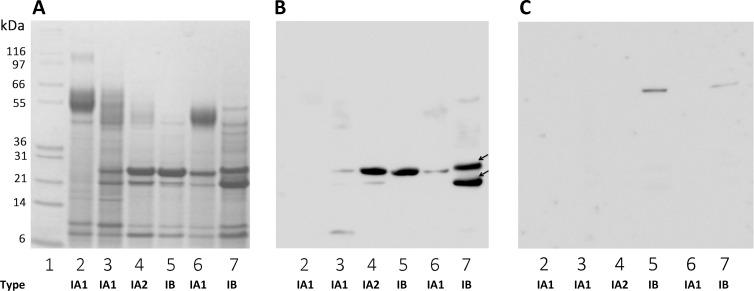
TLR2 binding to *P*. *acnes* surface proteins. *P*. *acnes* surface proteins were extracted from a 5-day culture bacterial pellet and separated by electrophoresis in a 4–12% NuPAGE LDS BisTris gel (50 μg) with detection by Coomassie blue staining (A). Separated proteins were transferred onto nitrocellulose membranes, which were incubated with recombinant TLR2 (B) and TLR4 (C) (0.1 μg/ml). TLR binding activity was detected with specific biotinylated antibodies against TLR2 and TLR4, respectively, as described in the Materials and Methods. Lane 1 contains the molecular mass markers. Lanes 2 to 7 contain proteins from strains 6919, RON, CHR, GUE, TRI, and PIE, respectively. Arrows indicate the positions of the 24.5- and 27.5-kDa bands of interest.

We investigated the protein recognized by TLR2, by extracting proteins in the presence of a high concentration of lithium, to ensure the selective removal of *P*. *acnes* surface proteins. We checked that all the strains used in this study were able to induce proinflammatory responses, by measuring CXCL8 production by keratinocytes, and also that the HaCaT keratinocyte cell line used in the *in vitro* assay could produce TLR2. Total cell lysates were analyzed by western blotting, which revealed the presence of a protein with a molecular weight of about 90-kDa in the HaCaT lysate, and in the 293T and ThP1 lysates used as positive controls (S1 Fig). The keratinocytes were then stimulated with both whole bacteria and lithium protein extract (LiE), and CXCL8 production was measured after 18 h of stimulation. Representative results are shown for the evaluation of the CXCL8 production from the 27 *P*. *acnes* strains used in this study (Fig 1). Whole *P*. *acnes* bacteria induced CXCL8 mRNA and protein production in a dose-dependent manner, at a MOI of 10 to 1000 (Fig 1A and 1B). LiE from the corresponding bacteria were tested and shown to have a similar capacity to induce CXCL8 mRNA and protein production in a dose-dependent manner (Fig 2A and 2B). Both whole bacteria and LiE of CHR (type IA_2_) and PIE (type IB) strains, and, to a lesser extent, RON (type IA_1_) strains, induced large amounts of CXCL8 at the lower MOI of 10 (Fig 1B) or for a LiE protein content of 3.1 μg/ml, whereas the other strains did not (Fig 2B). These results indicate that *P*. *acnes* surface proteins induce the inflammatory reaction and are a suitable starting material for identification of the surface protein recognized by TLR2. Far-western blotting was performed on total LiE, and two protein bands recognized by TLR2, with apparent molecular masses of 24.5- and 27.5-kDa, were detected (Fig 3B). According to the number of protein bands recognized by TLR2, and the intensity of the signal, we ranked strains as follows: (+++) for 2 strongly recognized bands (Fig 3B, lane 7); (++) for 2 moderately recognized bands (lane 4); (+) 1 moderately recognized band (lane 6); (+/-) 1 very faint band (lane 3); and (-)no band recognized by TLR2. The results for the 27 strains are presented in S2 Fig. No bands were detected in negative control experiments using only recombinant TLR2/detection substrate or biotinylated anti-TLR2 antibody/detection substrate (data not shown). We assessed the specificity of recognition by TLR2, by incubating the surface proteins with recombinant TLR4; only two faint bands were detected, at an apparent molecular weight of about 60-kDa (Fig 3C, lanes 5, 7) in only two strains, and neither the 24.5-kDa nor the 27.5-kDa protein was recognized by TLR4. As the 24.5-kDa and 27.5-kDa proteins appeared to be recognized by TLR2 in 48% of the strains tested, we chose to focus on the characterization of these two proteins.

### Proinflammatory activity of the proteins of interest

**Fig 4:**
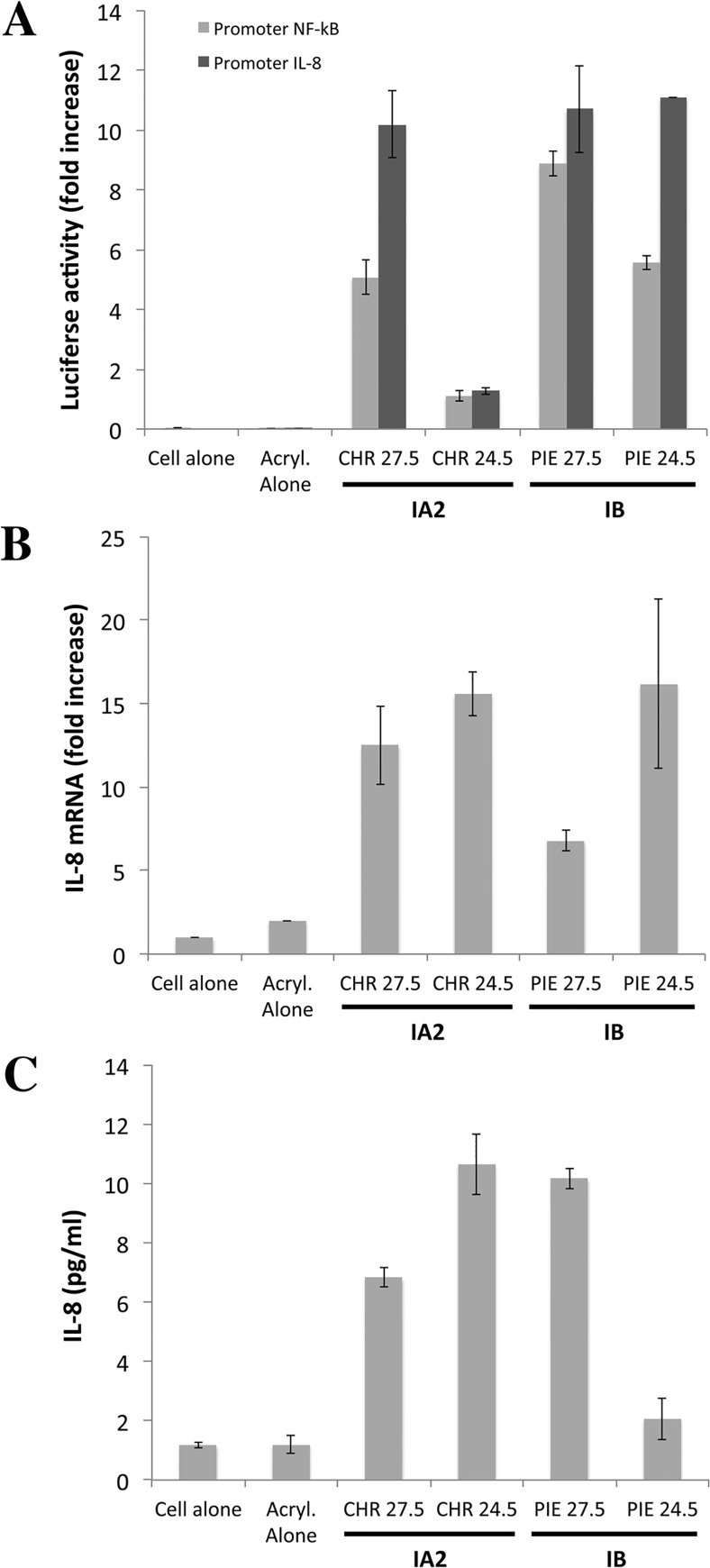
*P*. *acnes* surface proteins recognized by TLR2 have proinflammatory activity *in vitro*. Surface protein extracts were electrophoretically separated in 10% SDS-PAGE gels (13 x 13 cm), with detection by Coomassie blue staining. Proteins of interest were excised from the gel and eluted as described in the Materials and Methods. (A) HaCaT cells were transfected with CXCL8 (-173 bp) (light gray bar) and NF-κB (dark gray bar) inserted into a construct upstream from the luciferase gene, for 24 h, after which, an internal control (the *Renilla* luciferase expression plasmid) was added to the transfection mixture. Cells were stimulated by incubation with the eluted proteins (10 μg) for 24 h at 37°C. Relative NF-κB and CXCL8 promoter activities were determined as the ratio of firefly and *Renilla* luciferase activities. HaCaT cells were stimulated with the eluted proteins (20 μg) for 24 h at 37°C and CXCL8 mRNA levels were evaluated by RT-qPCR (B), and CXCL8 protein levels were assessed by ELISA (C). Data are presented as the mean ± standard deviation of two independent experiments. Statistical significance is indicated by * *P* ≤ 0.05, ** *P* ≤ 0.01, *** *P* ≤ 0.001, **** *P* ≤ 0.0001.

We assessed the proinflammatory properties of the 24.5- and 27.5-kDa proteins, by electrophoretic separation of these proteins from the LiE of the CHR (type IA_2_) and PIE (type IB) strains, elution from the gel and assessment of their ability to induce CXCL8 production (Fig 4). We showed in NF-κB and CXCL8 promoter assays that these proteins were able to activate both promoters (Fig 4A). The direct stimulation of epidermal keratinocytes by these two proteins also led to the 10- to 100-fold induction of CXCL8 transcription (Fig 4B) and an increase in the amount of CXCL8 protein to 10 pg/ml (Fig 4C). Control experiments were performed on cells alone and on cells stimulated with a solution eluted from the acrylamide gel in the same conditions; no activity was observed in either case. Both proteins of interest were able to stimulate the production of CXCL8 mRNA and protein in keratinocytes.

### Characterization of the proteins of interest

**Fig 5:**
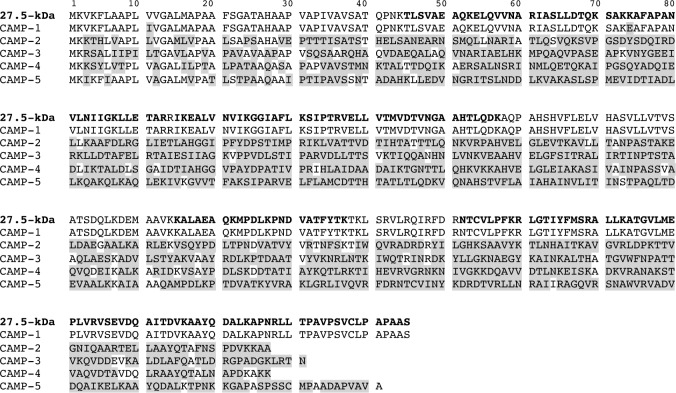
Comparison of the sequences of the 27.5 kDa *P*. *acnes* surface protein and *P*. *acnes* CAMP factors. The peptide sequence of the 27.5 kDa protein was obtained after LC-MS/MS analysis corresponding to 66% coverage (in bold), as described in the Materials and Methods. It was compared with the sequences of *P*. *acnes* CAMP factors 1 to 5 (Reference strain NCTC 737, GenBank accession number AY527218.1). Differences between sequences are highlighted in gray.

**Fig 6:**
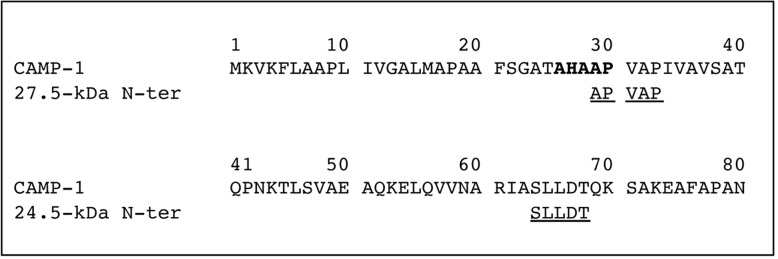
N-terminal sequencing of the protein of interest. Surface protein extracts were electrophoretically separated in 10% SDS-PAGE gels (13 x 13 cm), with detection by Coomassie blue staining. Proteins of interest were excised from the gel, eluted and subjected to N-terminal sequencing by Edman degradation to obtain the first five amino-acid residues of each protein, as described in Materials and Methods. N-terminal sequences were compared to the *P*. *acnes* CAMP factor 1 sequence (reference stain NCTC 737, GenBank accession number AY527218.1). The peptide signal cleavage site of CAMP factor 1 is shown in bold.

After electrophoretic separation, the proteins of interest were excised from the gel and characterized by LC-MS/MS of the peptide mixture obtained after in-gel digestion. Protein sequence database searches identified the two proteins of interest as products of gene encoding a hypothetical 285-amino acid protein from the CAMP factor superfamily (Accession number gi|488487765), covering 66% of the amino-acid sequence for the 27.5-kDa protein and 61% of the sequence for the 24.5-kDa protein (Table 1). The sequence of the 27.5-kDa was 99%, 38%, 33%, 50% and 51% identical to the *P*. *acnes* CAMP factors 1, 2, 3, 4, and 5, respectively (Fig 5), and both proteins corresponded to the same *P*. *acnes* surface protein. The theoretical molecular mass of CAMP factor 1 is 30.411-kDa. We therefore analyzed the N-terminal sequences of the two eluted proteins (Fig 6), comparing them with the expected sequence for CAMP factor 1. The first five amino acids of the 27.5-kDa protein were APVAP, located at the putative signal sequence cleavage site starting at position 29 [35]. This sequence is consistent with the loss of 2476 Da from the N-terminus of the protein, resulting in a truncated protein with a calculated molecular mass of 27.935-kDa. For the 24.5-kDa CAMP-1, the first five amino acids were SLLDT, at position 64, corresponding to the loss of 6322 Da, resulting in a truncated protein with a calculated molecular mass of 24.089-kDa (Fig 6). Based on these findings, we concluded that the surface protein of *P*. *acnes* recognized by TLR2 was CAMP factor 1.

**Table 1:** Proteins identified by LC-MS/MS in the trypsin-digested fragment from the 24.5- and 27.5-kDa *P*. *acnes* protein recognized by TLR-2

Protein band^a^	Accession number	Name	Score^b^	MW (kDa)	pI	Matched peptide	Sequence coverage (%)^c^
27.5	gi|488487765	MULTISPECIES: hypothetical protein [Propionibacterium]	1159	30379	9.88	28	66
	gi|488472745	MULTISPECIES: hypothetical protein [Propionibacterium]	1070	30394	9.77	25	66
	gi|495875308	hypothetical protein [Propionibacterium sp. 5_U_42AFAA]	1010	30408	9.77	23	64
	gi|488491325	hypothetical protein [Propionibacterium acnes]	967	30448	9.81	25	66
	gi|695301769	CAMP factor [Propionibacterium acnes]	813	30257	9.74	19	49
	gi|56244570	Camp4, partial [Propionibacterium acnes]	446	27466	9.4	10	52
	gi|313823890	hypothetical protein HMPREF9605_00761, partial [Propionibacterium acnes HL036PA2]	245	35838	10.04	6	32
	gi|313763866	Tat pathway signal sequence domain protein [Propionibacterium acnes HL013PA1]	71	32585	9.83	1	12
	gi|455647494	hypothetical protein H114_24280 [Streptomyces gancidicus BKS 13–15]	56	28817	5.4	1	2
24.5	gi|488487765	MULTISPECIES: hypothetical protein [Propionibacterium]	1159	30379	9.88	32	61
	gi|488472745	MULTISPECIES: hypothetical protein [Propionibacterium]	1058	30394	9.77	28	61
	gi|488491325	hypothetical protein [Propionibacterium acnes]	984	30448	9.81	27	61
	gi|495875308	hypothetical protein [Propionibacterium sp. 5_U_42AFAA]	952	30408	9.77	25	59
	gi|695301769	CAMP factor [Propionibacterium acnes]	705	30257	9.74	16	42
	gi|328907812	cAMP factor [Propionibacterium humerusii P08]	302	27322	9.7	6	24
	gi|663150685	transport integral membrane protein [Streptomyces violaceorubidus]	60	62204	9.8	0	1
	gi|492266975	phasin family protein [Acidovorax delafieldii]	55	18911	6.3	1	19
	gi|746357861	hypothetical protein, partial [Pandoraea sputorum]	53	160357	6.07	1	0

^a^ Electrophoretically separated protein bands were excised and subjected to in-gel trypsine digestion followed by LC-MS/MS analysis as described in Materials and Methods.

^b^ MASCOT score.

^c^ Coverage of the protein sequence by the peptides used for identification.

### Blocking of CAMP-1 / TLR-2 interaction

**Fig 7:**
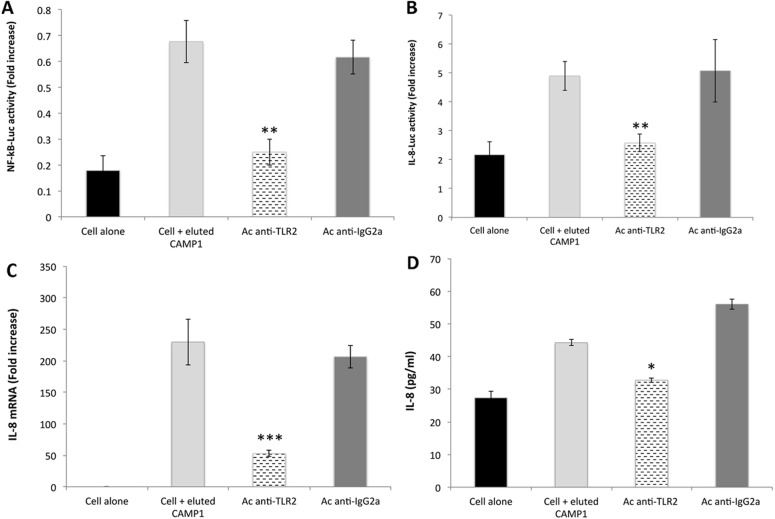
TLR2 blockade inhibits *P*. *acnes* CAMP factor 1-induced CXCL8 expression. Surface protein extracts were electrophoretically separated in 10% SDS-PAGE gels (13 x 13 cm), with detection by Coomassie blue staining. Proteins of interest were excised from the gel, eluted and pooled as described in the Materials and Methods. HaCaT cells were treated for 2 h with human anti-TLR2 (dashed bar) or with goat anti-IgGa antibodies (dark gray bar) and transfected by incubation for 24 h with NF-κB transcription factor (A) and CXCL8 (-173 bp) (B) inserted into a construct upstream from the luciferase gene, after which, an internal control (the *Renilla* luciferase expression plasmid) was added to the transfection mixture. Cell were stimulated with eluted CAMP factor 1 (50 μg/ml) for 24 h at 37°C. Relative NF-κB and CXCL8 promoter activities were determined as the ratio of firefly and *Renilla* luciferase activities. Pretreated HaCaT cells were stimulated by incubation with eluted CAMP factor 1 (50 μg/ml) for 24 h at 37°C, CXCL8 mRNA levels were determined by RT-qPCR (C), and CXCL8 production was measured by ELISA (D). Control experiments were performed with cells alone (dark bar) and with stimulated cells not previously treated with CAMP factor 1 (light gray bar). Data are presented as the mean ± standard deviation of two independent experiments. Statistical significance is indicated by * *P* ≤ 0.05, ** *P* ≤ 0.01, *** *P* ≤ 0.001, **** *P* ≤ 0.0001.

We investigated the ability of a blocking agent to outcompete the interaction of CAMP factor 1 with TLR2. Anti-TLR2 antibodies have been shown to block CXCL8 production by keratinocytes [18, 21]. The stimulation of keratinocytes with eluted CAMP-1 protein after their prior treatment with anti-TLR2 antibodies resulted in significantly lower levels of NF-κB (*P* = 0.008) and CXCL8 (*P* = 0.0012) promoter activation (Fig 7A and 7B), and, consequently, significantly lower levels of CXCL8 mRNA (*P* = 0.00047) (Fig 7C) and CXCL8 protein production in keratinocytes (*P* = 0.0024) (Fig 7D). No such effect was observed when this experiment was performed with the IgG isotype control. Thus, CAMP factor 1 isolated from *P*. *acnes* interacts with TLR2, triggering a downstream signaling pathway leading to CXCL8 production.

### Comparison of phylotype, TLR2 binding activity and CXCL8 production

Using a previously described rapid multiplex PCR assay [36], we phylotyped all the isolates. We identified four different phylotypes: IA_1_ (15 strains), IA_2_ (2 strains), IB (9 strains) and II (2 strains) (Table 2, S3 Fig). We analyzed the recognition of CAMP factor 1 by TLR2 and found that most of the strains with strong TLR2 binding activity were of types IB and II (Table 2). We then measured CXCL8 production by keratinocytes stimulated with the 27 strains. We found that the *P*. *acnes* strains tested could be separated into two groups, one with low to moderate levels of CXCL8 production (93 to 400 pg/ml) and the other with high levels of CXCL8 production (>400 pg/ml) (Table 2). Most of the strains producing high level of CXCL8 were of types IB and II. A comparison of phylotype with ability to induce inflammation for the 27 *P*. *acnes* strains studied showed that most of the strains with high levels of CAMP1-TLR2 binding activity belonged to types IB and II, whereas the type IA_1_ and IA_2_ strains produced CAMP factor 1 molecules that were only moderately strongly recognized, if at all, by TLR2 (Table 2).

**Table 2:** Comparison of *P*. *acnes* inflammatory capabilities with phylogroup and CAMP factor 1 gene nucleotide sequence

Strain ID	Type^a^	TLR2 binding^b^	CXCL8 production (pg/ml)^c^	Nucleotide sequence^d^			Amino acid sequence^e^		
				Identity^f^ (%)	No. of nucleotide differences relative to reference sequence		Identity^f^ (%)	No. of peptide differences relative to reference sequence	
75150	IA_1_	-	382 ± 48	100	0		100	0	
16351	IA_1_	-	233 ± 23	100	0		100	0	
17248	IA_1_	-	252 ± 43	100	0		100	0	
53468	IA_1_	-	255 ± 26	100	0		100	0	
41103	IA_1_	-	367 ± 24	100	0		100	0	
A24	IA_1_	-	232 ± 14	100	0		100	0	
A26	IA_1_	-	309 ± 12	100	0		100	0	
6919	IA_1_	-	318 ± 58	100	0		100	0	
78910	IA_2_	-	151 ± 45	100	0		100	0	
A44	IA_1_	-	242 ± 12	99	1	(G361>T)	99	1	(V121>L)
A30	IA_1_	++	290 ± 27	99	1	(A31>G)	99	1	(I11>V)
38862	IA_1_	-	366 ± 6	99	2	(A31>G; A319>G)	99	2	(I11>V; I107>V)
RON	IA_1_	+/-	413 ± 46	99	2	(A31>G; A319>G)	99	2	(I11>V; I107>V)
TRI	IA_1_	+/-	242 ± 58	99	2	(A31>G; A319>G)	99	2	(I11>V; I107>V)
22197	IA_1_	+	291 ± 47	99	2	(A31>G; A319>G)	99	2	(I11>V; I107>V)
CHR	IA_2_	++	590 ± 74	99	2	(A31>G; G221>A)	99	2	(I11>V; E74>K)
14230	IB	+++	93 ± 5	99	2	(A31>G; G220>A)	99	2	(I11>V; E74>K)
12513	IB	+++	591 ± 15	99	2	(A31>G; G220>A)	99	2	(I11>V; E74>K)
22795	IB	+/-	457 ± 12	99	2	(A31>G; G220>A)	99	2	(I11>V; E74>K)
47474	IB	+++	314 ± 53	99	2	(A31>G; G220>A)	99	2	(I11>V; E74>K)
27387	IB	+++	259 ± 8	99	2	(A31>G; G220>A)	99	2	(I11>V; E74>K)
25236	IB	+++	267 ± 30	99	2	(A31>G; G220>A)	99	2	(I11>V; E74>K)
A9	IB	+++	310 ± 24	99	2	(A31>G; G220>A)	99	2	(I11>V; E74>K)
GUE	IB	+++	262 ± 75	99	2	(A31>G; G220>A)	99	2	(I11>V; E74>K)
PIE	IB	++	447 ± 55	99	2	(A31>G; G220>A)	99	2	(I11>V; E74>K)
27647	II	++	603 ± 26	96	36	(A31>G; A102>G; C163>T; A222>G; G231>A; C241>A; T242>C; T249>C; C258>T; G267>C; G278>A; G304>A; G306>T; C309>T; A319>G; T324>C; T330>G; C384>T; C387>T; T400>C; C450>T; A465>G; A472>G; C556>A; A561>G; T572>G; A592>G; T601>G; T606>C; C642>T; A679>T; T705>C; T813>C; T826>G; C835>G; T836>C)	96	14	(I11>V; I34>M; L82>T; R93>K; V102>I; I107>V; T158>A; L186>I; V191>G; T198>A; S201>A; M226>L; S276>A; L279>A)
A11	II	+++	569 ± 41	96	36	(A31>G; A102>G; C163>T; A222>G; G231>A; C241>A; T242>C; T249>C; C258>T; G267>C; G278>A; G304>A; G306>T; C309>T; A319>G; T324>C; T330>G; C384>T; C387>T; T400>C; C450>T; A465>G; A472>G; C556>A; A561>G; T572>G; A592>G; T601>G; T606>C; C642>T; A679>T; T705>C; T813>C; T826>G; C835>G; T836>C)	96	14	(I11>V; I34>M; L82>T; R93>K; V102>I; I107>V; T158>A; L186>I; V191>G; T198>A; S201>A; M226>L; S276>A; L279>A)

^a^: Phylotyping was performed by the multiplex method [3].

^b^: TLR2 binding to CAMP factor 1 was assessed by Far-western blotting, as described in the Materials and Methods.

^c^: CXCL8 production was measured by ELISA on the culture supernatant from HaCaT keratinocytes stimulated with whole bacteria at a MOI of 10.

^d^: The CAMP factor 1 gene was amplified as described in the Materials and Methods and sequenced with ABI PRISM Ready Reaction Terminator cycle sequencing kits, as described in the Materials and Methods. All sequences are compared to the reference sequence of *P*. *acnes* strain NTCT 737.

^e^: The amino-acid sequence of CAMP factor 1 was deduced with AliView software.

^f^: The identity between CAMP factor 1 sequences was assessed by BLAST analysis.

### CAMP-1 nucleotide and peptide sequences analysis

**Fig 8:**
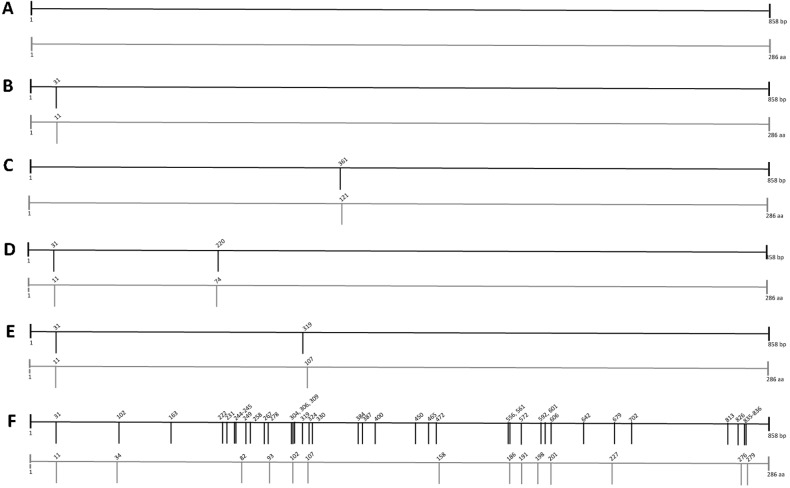
*P*. *acnes* CAMP factor 1 polymorphism. CAMP factor 1 nucleotide sequence analysis was performed on the 27 *P*. *acnes* isolates, as described in the Materials and Methods. Nucleotide (dark bold) and peptide (light gray) changes identified in *P*. *acnes* isolates relative to the reference sequence of the *P*. *acnes* NTCT 737 strain (GenBank accession number AY527218.1) are indicated by vertical bars. (A), (B and C), (D and E), (F) correspond to CAMP1 with no, 1, 2, and several mutations, respectively. See also Table 4.

CAMP1-TLR2 binding profile differed between *P*. *acnes* strains (Fig 3). We therefore investigated the nucleotide and peptide sequences of the 27 *P*. *acnes* strains. The 858 bp corresponding to the sequence of CAMP factor 1 reported elsewhere [35] was sequenced and compared with the sequence obtained for CAMP factor 1 reference strain NCTC 737 (ATCC 6919). Sequences were then classified into four groups according to the number of nucleotide polymorphisms (Fig 8, Table 2). The first group corresponded to CAMP factor 1 genes with no nucleotide polymorphism (Fig 8A). The second group corresponded to CAMP factor 1 genes with 1 nucleotide polymorphism, either A31>G or G361>T, corresponding to amino-acid changes at positions I11>V and V121>L, respectively (Fig 8B and 8C; Table 2). A third group consisted of CAMP factor 1 genes displaying two single nucleotide polymorphisms from the set of A31>G, G220>A and A319>G, corresponding to the following changes in amino-acid sequence: I11>V, E74>K and I107>V, respectively (Fig 8D and 8E; Table 2). The final group corresponded to CAMP factor 1 genes with sequences containing 36 single nucleotide polymorphisms, A31>G, A102>G, C163>T, A222>G, G231>A, C241>A, T242>C, T249>C, C258>T, G267>C, G278>A, G304>A, G306>T, C309>T, A319>G, T324>C, T330>G, C384>T, C387>T, T400>C, C450>T, A465>G, A472>G, C556>A, A561>G, T572>G, A592>G, T601>G, T606>C, C642>T, A679>T, T702>C, T813>C, T826>G, C835>G, T836>C, corresponding to 14 amino-acid changes: I11>V, I34>M, L82>T, R93>K, V102>I, I107>V, T158>A, L186>I, V191>G, T198>A, S201>A, M226>L, S276>A, L279>A (Fig 8F; Table 2). The relationship between CAMP-1 sequences and CAMP factor 1-TLR2 binding activities was investigated for the 27 *P*. *acnes* strains. Strains with weaker TLR2 binding activity were found to have CAMP factor 1 genes with no nucleotide polymorphism or sequences with 1 or 2 nucleotide polymorphisms, corresponding to I11>V, I107>V and V121>L. Conversely, *P*. *acnes* strains with strong CAMP1-TLR2 binding activity had CAMP1 sequences with nucleotide polymorphisms resulting in I11>V and E74>K substitutions or had large numbers of nucleotide polymorphisms, resulting in 14 amino-acid changes (Table 2).

### Phylogenetic analysis for CAMP factor 1

**Fig 9:**
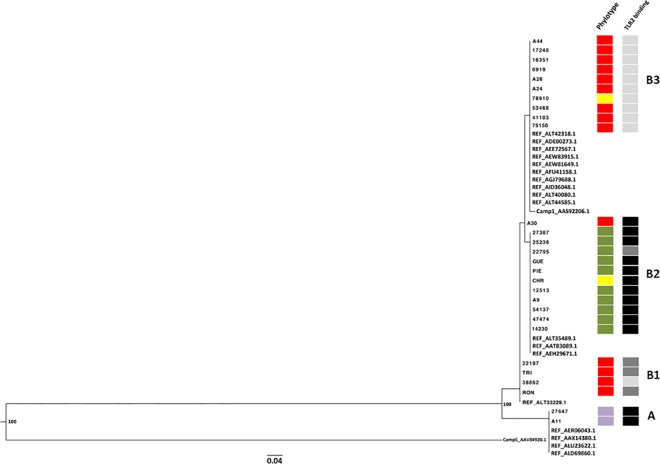
Phylogenetic analysis of the relationship between *P*. *acnes* CAMP factor 1 proteins. Phylogenetic analysis illustrating the relationship between CAMP factor 1 and 19 protein reference sequences from *P*. *acnes* (GenBank accession numbers ALT42318.1, ADE00273.1, AEE72567.1, AEW83915.1, AEW81649.1, AFU41158.1, AGJ79688.1, AID36048.1, ALT40080.1, ALT44585.1, AAS92206.1, ALT35489.1, AAT83098.1, AEH29671.1, ALT33229.1, AER06043.1, AAX14380.1, ALU23622.1, ALD69860.1), CAMP 5 factor Protein (AAV84920.1) and sequences of the 27 *P*. *acnes* strains used in this study. Phylogenetic trees were constructed by the maximum likelihood method, using PhyML3.0 [65], and the tree was rooted on the CAMP factor 5 protein sequence. Bootstrapping was applied to the trees (500 datasets) and bootstrap values are shown at nodes. The bar indicates the number of substitutions per site. Sequences were classified into four groups (A, B1 to B3). Squares correspond to phylotype IA_1_ (red), IA_2_ (yellow), IB (green), II (purple); and to CAMP1-TLR2 binding intensity with no binding as—(light gray), weak binding as +/- and + (dark gray), strong binding as ++ and +++ (black).

Phylogenetic analysis of the CAMP factor 1 sequences revealed the existence of two distinct genetic groups supported by high bootstrap values, and corresponding to strains phylotyped II (27647, A11) with CAMP factor 1 containing 36 nucleotide polymorphisms (group A), and to the strains phylotyped IA_1_, IA_2_, IB with CAMP factor 1 containing no nucleotide polymorphism (17248, 16351, 6919, A26, A24, 78910, 53468, 41103, 75150), one nucleotide polymorphism (A44), and 2 nucleotide polymorphisms (RON, 38862, TRI, 22197, 27387, 25236, 22795, GUE, PIE, CHR, 12513, A9, 47474, 14230) (Group B) (Fig 9; Table 2). All CAMP factor 1 sequences other than that from isolate A30 clustered together. Group B contained three genetically related clusters (B1, B2, B3) corresponding to different intensities of TLR2 binding. Cluster B1 corresponded to CAMP factor 1 molecules very weakly recognized by TLR2 (RON, 38862, TRI, 22197). Cluster B2 corresponded to CAMP factor 1 molecules strongly recognized by TLR2 (27387, 25236, 22795, GUE, PIE, CHR, 12513, A9, 47474, 14230); and cluster B3 corresponded to CAMP factor 1 molecules not recognized by TLR2 (A44, 17248, 16351, 6919, A26, A24, 78910, 53468, 41103, 75150) (Fig 9B).

## Discussion

The aim of this study was to characterize the *P*. *acnes* surface protein recognized by TLR2. We used keratinocytes in our *in vitro* assay, because these cells are the first line of defense against external aggressions. They contribute to the innate immune response by producing chemotactic factors that attract leukocytes and neutrophils to the site of infection [25]. *P*. *acnes* has been shown to trigger the production of proinflammatory molecules by monocytes [28, 33,], and the massive secretion of IL-1α, TNF-α [26], IL-8 [37], and reactive oxygen species from keratinocytes [29]. In this study, we used the HaCaT cell line, corresponding to spontaneously transformed human adult skin keratinocytes. These cells can differentiate [38], express Toll-like receptors [39] and produce numerous cytokines in response to bacterial stimulation [27]. We found that all strains induced CXCL8 production *in vitro* in a dose-dependent manner, although the amount of CXCL8 produced differed between strains. This finding is consistent with previous studies describing differences in the modulation of the immune response between different *P*. *acnes* strains [12, 21]. We assessed the ability of surface proteins to induce CXCL8 production *in vitro*. All *P*. *acnes* crude surface protein extracts were able to activate the synthesis of CXCL8 mRNA and protein by keratinocytes, and were therefore considered suitable for further analysis. The overproduction of CXCL8 observed with whole bacteria was reproduced with the corresponding protein extract, consistent with the presence of specific molecular material involved in CXCL8 induction. However, overall CXCL8 levels were lower for protein extracts than for whole bacteria, suggesting a partial loss of proteins during the extraction process. Using Far-western blot analysis, we characterized two surface proteins recognized by TLR2 with apparent molecular masses of 24.5- and 27.5-kDa; no bands were detected in the control experiments. We assessed the specificity of recognition, by testing the same protein extracts with recombinant TLR4. No cross-reactivity was observed, suggesting that the interaction with TLR2 was specific. For confirmation of the relevance of the 24.5- and 28.5-kDa proteins as PAMP candidates, we evaluated the ability of these proteins, isolated from electrophoresis gels, to induce inflammation in keratinocytes. Both proteins activated the CXCL8 and NF-κB promoters, and induced the production of CXCL8 mRNA and protein. These results are consistent with these two proteins being good candidates for triggering an inflammatory response. These proteins were then subjected to proteomic analysis and characterized as *P*. *acnes* CAMP factor 1. CAMP is the acronym for the Christie-Atkins-Munch-Petersen reaction, a co-hemolytic reaction of erythrocytes involving synergy between the CAMP protein from group B streptococci and the sphingomyelinase (SMase) from *S*. *aureus* [40]. CAMP factors have been identified in several bacterial species, including *Streptococcus agalactiae* [41], and *Pasteurella haemolytica* [42]. Our results are consistent with those of previous studies reporting a co-hemolytic reaction involving *P*. *acnes* [43], and the identification of nucleotide sequences for *P*. *acnes* CAMP factors 1–5 in whole genomes and the detection of the corresponding proteins in protein extracts [35, 44,].

In this study, two proteins were identified as binding to TLR2, and these two proteins matched two proteins stained with Coomassie blue on the gel. A previous study detected only one protein, at about 28-kDa, with a monoclonal antibody against CAMP factor 1 and staining [35]. This discrepancy may be accounted for by the detection methods (antibody versus TLR2 binding), growth media (BHI versus RCM), and protein extraction methods (sonication/detergent versus lithium) used. According to the peptide sequence of CAMP factor 1 from the reference strain NCTC 737, which contains 285 amino-acid residues, the calculated molecular mass of the protein is 30.411-kDa including the signal sequence; the removal of the signal sequence would result in a protein of 27.656-kDa [35]. In terms of precision, we considered the two proteins at 28-kDa [35] and 27.5-kDa to correspond to CAMP factor 1 without its signal sequence. This hypothesis was confirmed by N-terminal sequencing of the 27.5-kDa protein, which yielded a sequence of APVAP. However, for the 24.5-kDa protein, which was also identified as CAMP factor 1, the N-terminal sequencing obtained was SLLDT. No other putative cleavage site has ever been described and additional studies will be required to determine whether this truncated CAMP factor 1 protein originates from *P*. *acnes* itself, is produced artificially during the extraction process or results from effects of the growth medium on protein production, as shown in a previous study [14]. Moreover, although CXCL8 induction experiments have shown that both the full-length and truncated forms of CAMP factor 1 are active, it is not yet possible to draw any firm conclusions about which of these forms is most active.

There is currently no clear evidence concerning the fate of *P*. *acnes* CAMP factors: secreted and/or associated with the cell surface. Previous studies of CAMP factors in *S*. *agalactiae* showed these factors to be both present on the bacterial surface and secreted into the culture medium [45]. In this study, we extracted surface proteins from washed bacterial pellets by lithium stripping. Our results are consistent with the CAMP factor 1 protein being associated with the surface but not firmly bound to it, as it can be removed from whole bacteria by trypsin cleavage [46]. Previous studies have reported the presence of *P*. *acnes* CAMP factors in both the external medium and on the cell surface, but with considerable differences between strains [22, 35]. However, growth phase conditions have been shown to influence the expression of *P*. *acnes* genes [47]. In our study, we systematically used the same growth anaerobic conditions, until stationary phase was reached (OD > 0.6), on reinforced clostridial media. CAMP factors have no C-terminal LPXTG anchoring domain. Further investigations will therefore be necessary to determine how CAMP factor 1 is produced and processed at the surface of *P*. *acnes* and secreted into the external medium.

Nothing is currently known about the recognition of CAMP proteins by TLRs. CAMP factors can bind to several proteins, including immunoglobulins, via their Fc-binding region [48], and GPI-anchored proteins, via their glycan moieties [49]. TLR2 recognition preferentially involves lipoproteins expressed at the bacterial surface, but several members of the no-lipoprotein pore-forming toxin family have been shown to interact with TLRs, consistent with broad specificity for TLR2-mediated recognition [50]. Further studies are underway to identify the parts of the protein involved in this binding.

In this study, four phylotypes were identified. Most of the acne- (4/6) and skin-related strains (3/4) belonged to type IA_1_, whereas the strains isolated from bone, joint and soft tissue infections mostly belonged to phylotypes IA_2_, IB and II. This result is consistent with previous findings [23]. We found that the intensity of TLR2 binding to CAMP factor 1 differed between the strains tested. Indeed, 59% (16/27) of the strains displaying little or no TLR2-CAMP1 binding activity were phylotyped IA_1_ and IA_2_, whereas strains with a strong binding activity were typed IB and II. Moreover, the levels of CXCL8 production induced by *P*. *acnes* in keratinocytes seemed to be higher for the IB and II phylotypes, and lower for the IAs phylotype. This difference in CAMP1-TLR2 binding activity may reflect two phenomena: 1) differential CAMP1 gene expression and, 2) CAMP1 sequence variation affecting binding activity. Differences in CAMP factor 1 gene expression between *P*. *acnes* strains have been described before, with CAMP1 genes most strongly expressed in types IB and II, but analyses of the Shine-Dalgarno sequence shed no light on the reasons for this [35]. Many bacteria regulate virulence factor expression as a function of growth phase. Indeed, *P*. *acnes* produces most of its secreted virulence-associated factors during the exponential growth phase [47], and we cannot exclude the possibility of a role for regulatory proteins in modulating CAMP factor 1 production, as previously reported for the major carbon catabolite repressor protein CcpA, which regulates expression of the CAMP gene in *Streptococcus pyogenes* by interacting directly with its promoter [51]. We first investigated possible variations of the CAMP factor 1 sequences from the 27 strains, and detected considerable genetic polymorphism. This result is consistent with previous studies reporting differences in CAMP factor nucleotide and amino-acid sequences between *P*. *acnes* phylotypes [18]. We found that CAMP factor 1 was genetically distant from the other CAMP factors, but more related to CAMP factor 5, as previously described [35]. CAMP factor 1 sequences formed two main groups, according to the level of sequence polymorphism. The first group (A) was characterized by a highly polymorphic CAMP-1 sequence, with 14 amino-acid changes relative to the reference sequence, isolated from type II strains with moderate and high levels of TLR2 binding activity and with strong CXCL8 induction. This group included one strain (A11) isolated from a patient with severe acne. The other group (B) was more heterogeneous, and included CAMP sequences displaying 0, 1 or 2 amino-acid changes relative to the reference sequence. Interestingly, the group with no sequence polymorphism (B3) corresponded to strains inducing low or moderate levels of CXCL8 production, producing a CAMP factor 1 not recognized by TLR2; all these strains were isolated from patients with moderate acne. Otherwise, the groups presenting 1 or 2 sequence polymorphisms (B1, B2) corresponded to strains with a CAMP factor 1 recognized by TLR2 and inducing moderate or high levels of CXCL8 production. These results suggest that CAMP factor 1 recognition by TLR2 may be associated with the presence of polymorphisms in its sequence. Given that we have characterized a truncated CAMP-1 protein, it would be logical to rule out the I11>V amino-acid change as being of relevance. The I107>V polymorphism seems to be associated with low levels of TLR2 binding activity, whereas the E74>K polymorphism is present in CAMP-1 sequences from strains with high levels of TLR2 binding activity. Two strains did not seem to match this pattern (38862 and 22795) and may not be the best representative strains for their groups, because previous studies have shown that neither the type IB strain KPA171202 nor the type II strain NCTC 10390 produces CAMP factor 1 [35, 52,]. Furthermore, A30 was the only IA_1_ type strain tested to display moderate TLR2 binding and moderate CXCL8 induction. This strain was isolated from a patient with severe acne and appears to be genetically separate from the other groups. However, the production of small or moderate amounts of CXCL8 by keratinocytes in response to IA *P*. *acnes* strains indicates that other *P*. *acnes* surface molecules are involved in this process. Indeed, as in other Gram-positive bacteria, peptidoglycan is a major constituent of the outer envelope in *P*. *acnes* and it promotes CXCL8-related inflammation through the TLR2 pathway [28, 53,]. Overall, these results suggest that specific sequence polymorphisms may play an important role in determining the strength of CAMP1-TLR2 binding. However, this approach has limitations and further investigations with mutant strains lacking CAMP1 expression, and with recombinant forms of CAMP1 will be required for better evaluations of the intensity and the specificity of TLR2 recognition.

Several components of Gram-positive bacteria, such as peptidoglycan, lipoteichoic acid, and lipoproteins, have been shown to induce inflammation by interacting with TLR2. However, LPS-TLR4 interaction seems to induce a stronger inflammation response than PGN-TLR2 interaction [37], and the *P*. *acnes* inflammatory mediators able to induce reactions resembling those induced by LPS have not yet been identified. *P*. *acnes* was long considered a bacterial contaminant, but several studies have provided evidence to suggest that *P*. *acnes* should be considered an opportunistic pathogen producing several proteins potentially involved in the degradation of molecules present in host tissues, together with cell wall-associated surface proteins that may interact with the host, triggering inflammation [23, 54,]. CAMP factors are among the proteins thought to be involved in *P*. *acnes* pathogenesis [55, 56]. These factors are present in both commensal and pathogenic strains and their classification as “host adaptation factors” potentially helping the bacteria to survive in this highly competitive niche has been suggested, as *S*. *aureus* bacteria have been reported to use *P*. *acnes* CAMP factors to enhance their virulence [23,57,]. CAMP factor 1 is expressed by *P*. *acnes* in both case and control samples [58], but the injection of recombinant CAMP into mouse ear induces local inflammation [55]. CAMP factor 2 knockout partially alters co-hemolytic activity, whereas CAMP factor 4 knockout has no effect, suggesting redundancy between these factors. Moreover, the stimulation of HaCaT keratinocytes with a ΔCAMP2/4 knockout mutant revealed no differences in initial inflammatory response, whereas expression of the neutral SMase 3 gene was deregulated in the ΔCAMP4 mutant [52]. Moreover, the interaction of *P*. *acnes* CAMP factors with SMase suggests that CAMP factors may be involved in *P*. *acnes*-related pathogenesis [56]. In conclusion, we suggest that CAMP factor 1 may contribute to *P*. *acnes* virulence by amplifying the inflammation reaction through direct interaction with TLR2.

## Materials and Methods

### Bacterial culture and protein extraction

We used 27 strains of *P*. *acnes*: strain 6919 from the American Type Culture Collection (Manassas, VA), 6 strains from acne lesions (3 from patients with severe acne and 3 from patients with moderate acne, collected at the Dermatology Department of Nantes Hospital), and 21 strains isolated from various infections (11 bone and joint infections, 1 case of pleuritis, 1 of pericarditis, 2 of pneumonia, 2 cutaneous infections, 1 case of bacteremia, and 3 ascitic fluid infections) (Table 3). *P*. *acnes* strains were grown under anaerobic conditions in liquid reinforced clostridial medium (RCM) at 37°C for five days, until they reached stationary phase. Typically, cultures were carried out in 100 to 1000 ml of RCM and bacteria were harvested by centrifugation at 5 000 x *g* for 10 minutes at room temperature. Pellets were washed in 20 to 200 ml of PBS and centrifuged again. The total surface protein extract was obtained by suspending the bacterial pellet in 20 to 200 ml of 1 M LiCl and incubating at 4°C for 18 h with gentle mixing. The bacterial suspension was centrifuged at 5 000 x *g* for 20 minutes to remove the bacteria, and the supernatant, containing the surface proteins, was concentrated by precipitation with ammonium sulfate at 80% saturation for 18 h at 4°C. The precipitated proteins were recovered by centrifugation at 15 000 x *g* for 30 minutes at 4°C, resuspended in 20 ml of PBS and extensively dialyzed against PBS. Protein concentration was determined by the Lowry method, using BSA as the standard, as previously described [59].

**Table 3:** Characteristics of *P*. *acnes* strains

Strain ID	Location	Species^a^	Treatment or diagnosis
75150	Bone	*P*. *acnes*	yes
16351	Ascites	*P*. *acnes*	yes
17248	Ascites	*P*. *acnes*	none
53468	Lung	*P*. *acnes*	none
41103	Skin	*P*. *acnes*	none
A24	Skin (acne)	*P*. *acnes*	moderate acne
A26	Skin (acne)	*P*. *acnes*	moderate acne
6919	Skin	*P*. *acnes*	/
78910	Synovial fluid	*P*. *acnes*	none
A44	Skin (acne)	*P*. *acnes*	moderate acne
A30	Skin (acne)	*P*. *acnes*	severe acne
38862	Skin	*P*. *acnes*	none
RON	Bone	*P*. *acnes*	yes
TRI	Bone	*P*. *acnes*	yes
22197	Bone	*P*. *acnes*	yes
CHR	Bone	*P*. *acnes*	yes
14230	Bone	*P*. *acnes*	yes
12513	Pleural liquid	*P*. *acnes*	none
22795	Pericardium	*P*. *acnes*	yes
47474	Ascites	*P*. *acnes*	none
27387	Bone	*P*. *acnes*	yes
25236	Bone	*P*. *acnes*	yes
A9	Skin (acne)	*P*. *acnes*	severe acne
GUE	Bone	*P*. *acnes*	yes
PIE	Bone	*P*. *acnes*	yes
27647	Blood	*P*. *acnes*	none
A11	Skin (acne)	*P*. *acnes*	severe acne

^a^: *P*. *acnes* strains were identified by MALDI analysis

### Cell culture and stimulation

The human keratinocyte cell line HaCaT (provided by Dr N.E. Fuseining, Heidelberg, Germany) was grown in Dulbecco’s modified Eagle’s medium-Glutamax-I (DMEM) (Invitrogen, Cergy Pontoise, France). The ThP1 immortalized human monocytic cell line was grown in Roswell Park Memorial Institute 1640 Medium-Glutamax-I (RPMI). DMEM and RPMI were supplemented with 10% heat-inactivated fetal calf serum (Invitrogen, Cergy Pontoise, France), and an antibiotic/antimycotic solution (10 U/ml pencillin, 10 μg/ml streptomycin, 0.25 μg/ml amphotericin; Invitrogen) and cultures were incubated at 37°C, under a humidified atmosphere containing 5% CO_2_. For stimulation experiments, HaCaT cell were incubated with a *P*. *acnes* suspension adjusted to the appropriate concentration (by absorbance measurement at 620 nm) or with lithium protein extract (LiE) in buffer solution, for desired period of time, at 37°C, under an atmosphere containing 5% CO_2_.

### DNA extraction and phylotyping

Bacterial strains were grown for 5 days at 37°C under anaerobic conditions and DNA was extracted with the E.Z.N.A. ®Bacterial DNA kit, in accordance with the manufacturer’s recommendations (Omega Bio-tek, Norcross, USA). DNA concentration was determined by measuring absorbance at 260 nm (Nanodrop 2000 ThermoScientific). The values obtained were in the range of 30 to 220 ng/μl, and the concentration of the solution was adjusted as required. Phylotyping was performed by multiplex touchdown PCR, as previously described [3], and the amplification reaction was carried out with a mixture containing 0.2 U *Taq* DNA polymerase, 1X PCR buffer, 50 mM MgCl_2_, 5 or 10 mM dNTP, with an initial denaturation at 94°C for 60 s followed by 14 cycles of 94°C for 30 s, 66°C for 30 s, and 72°C for 60s; then 11 cycles of 94°C for 30 s, 62°C for 30 s and 72°C for 60 s. The mixture was then subjected to a final elongation step consisting of heating at 72°C for 10 minutes. The genes amplified were the 16S rRNA (expressed by all *P*. *acnes* phylogroups), ATPase (type IA_1_, IA_2_, IC), *sodA* (types IA_2_, IB), toxin (Fic family) (type IC), *atpD* (type II), and *recA* (type III) genes, with the primers described elsewhere [35]. The PCR products (5 μl) were analyzed by electrophoresis in a 1.5% agarose gel and the amplified nucleotide bands were visualized by UV transillumination of the ethidium bromide-stained gel, with comparison to a 50-bp DNA ladder (Invitrogen, Carlsbad, California, USA).

### ELISA

Human CXCL8 protein concentration was determined in the supernatants of cells stimulated with whole bacteria, LiE or eluted proteins, by ELISA, according to the kit manufacturer’s instructions (eBioscience). We used serial dilutions of recombinant human CXCL8 to generate the standard curve. Optical density was determined at 450 nm, with wavelength correction at 540 nm.

### Far-western blotting

*P*. *acnes* surface proteins (50 μg) were separated by electrophoresis (LDS-PAGE) under denaturing conditions, in a NuPAGE Novex 4–12% Bis-Tris gel (1 mm, 12 wells, Invitrogen, UK). Two gels were run simultaneously. One gel was stained with Coomassie blue for protein detection. The proteins separated in the second gel were transferred onto a nitrocellulose membrane, which was then saturated by incubation with 10 ml of saturation buffer consisting of 1X TBS (Tris-buffered saline) containing 200 mM Tris, 1.4 M NaCl (pH 7.6), 5% nonfat milk, and 0.1% Tween 20 for 1 h. The membrane was washed three times, for 15 minutes each, with 15 ml of TBS/T buffer [1X TBS, 0.1% Tween-20], then incubated overnight with 10 ml of human recombinant TLR2 (R&D Systems, Abingdon, UK) diluted to 0.1 μg/ml in TBS/T, at 4°C, with gentle mixing. Unbound antibodies were removed by washing as described above, and the membrane was incubated with biotinylated human antibodies against TLR2 or TLR4 (R&D Systems, Abingdon, UK) diluted to 0.1 μg/ml in TBS/T supplemented with 5% BSA, for 20 h at 4°C, with gentle mixing. The membrane was washed to remove unbound antibodies, and the surface protein-TLR2-biotinylated antibody complex was detected by incubation for 1 h at room temperature with HRP (horseradish peroxidase) diluted to 0.5 μg/ml in saturation buffer. Unbound material was removed by washing and peroxidase activity was detected in a chemiluminescence assay (WesternBright ECL, Advansta, Menlo Park, USA).

### Western blotting

HaCaT and ThP1 cells were washed in sterile cold PBS and lysed in RIPA buffer containing 50 mM Tris, 150 mM NaCl, 1% NP-40, 0.5% sodium deoxycholate, 0.1% SDS (pH 8.0). Total extracts were centrifuged at 14,000 x *g* for 15 minutes at 4°C and the supernatant was used as total protein extract. Protein samples (15 μg), and lysates of human TLR2-transfected 293T cells were fractionated by electrophoresis in a NuPAGE Novex 4–12% Bis-Tris gel (1 mm, 12 wells, Invitrogen, UK) and transferred onto nitrocellulose membranes. Membranes were saturated by incubation for 1 h at room temperature in TBS/T buffer and incubated at 4°C for 18 h with goat polyclonal IgG antibodies against human TLR2 (C-19), diluted to 0.5 μg/ml in TBS supplemented with 5% BSA, and 0.1% Tween-20 (all antibodies and 293T lysate were purchased from Santa Cruz Biotechnology, Inc., Santa Cruz, Calif., USA). The membranes were washed and bound antibodies were detected by incubation with donkey polyclonal antibodies against goat IgG coupled to peroxidase, diluted 1:2000 in saturation buffer, for 1 h at room temperature. Unbound material was removed by washing and peroxidase activity was detected in a chemiluminescence assay (Advansta Corp., Menio Park, Calif. USA).

### Protein elution

Proteins were fractionated by electrophoresis in denaturing conditions (SDS-PAGE, gel 13 x 13 cm) and detected by Coomassie blue. Proteins from a second gel were transferred onto nitrocellulose membranes and analyzed by Far-western blotting, as described above. Proteins recognized by TLR2 were matched to the stained bands, and excised from the gel. Excised protein bands were incubated for 18 h at 37°C in the presence of elution buffer (1% SDS in PBS), with stirring. The excess of SDS was removed by precipitation of the proteins in cold acetone (vol/vol) and centrifugation for 30 minutes at 14 000 x *g* and 4°C. Protein pellets were suspended in PBS and protein concentration was determined by spectroscopy at 280 nm (NanoDrop).

### Cell transfection and luciferase reporter gene assay

HaCaT cells were cultured in 24-well plates (Corning Costar) with DMEM containing 0.1% FCS without antibiotics. They were then transfected with reporter plasmids containing the CXCL8 promoter (-173 bp) or the NF-κB promoter (5 units) upstream from the firefly luciferase gene in JetPEI^TM^ (Polypolus Transfection, Illkirch, France) [60]. Transfection efficiency was normalized by co-transfecting the cells with 10 ng of pSRa-*Renilla* luciferase expression vector. The cells were incubated for 24 h, and were left unstimulated or were stimulated with LiE or proteins of interest eluted from the gel after electrophoretic separation. After 18 h of incubation at 37°C, cells were washed with sterile cold PBS and scraped directly into the lysis buffer provided by the manufacturer (Promega Corp., Madison, WS, USA). Firefly and *Renilla* luciferase activities were measured on a MicroLumat Plus LB 96V Luminometer (Bertold Technologies, Bas Wild-Bad, Germany). Results are expressed as a fold-increase relative to the basal activity measured in unstimulated transfected cells.

### RNA isolation and RT-qPCR

HaCaT cells were grown in six-well plates (Corning Costar) for 24 h with 10% FCS in DMEM, and then for 24 h in 0.1% FCS DMEM. HaCaT cells were then incubated for 5 h at 37°C under an atmosphere containing 5% CO_2_ and stimulated with whole bacteria (MOI 10), LiE (50 μg/ml), or proteins of interest eluted after electrophoretic separation (50 μg/ml). After stimulation, cells were washed twice with cold sterile PBS and scraped into lysis buffer, according to the manufacturer’s instructions (Macherey-Nagel). Total RNA was isolated with the NucleoSpin® RNA II kit (Machery-Nagel), according to the manufacturer’s instructions. RNA concentration was determined at 260 nm (NanoDrop). It ranged from 110 to 225 ng/μl, and was adjusted to desired concentration. RT-qPCR was performed with the iTaq Universal Sybr Green one-Step kit (BioRad) containing reverse transcriptase (RT) and *Taq* polymerase. Complementary DNA was generated from 330 ng of total RNA by RT for 10 minutes at 50°C, in a final volume of 10 μl. Standard amplification for quantitative PCR was carried out in the same test tube containing the reaction mixture, and the PCR conditions were as follows: 95°C for 60 s, followed by 40 cycles of 95°C for 15s, 68°C for 60s, and 72°C for 15s, followed by a final stage to obtain a melting curve for 65 to 95°C, at 0.1°C/s over 60 s on a LightCycler Nano (Roche). PCR results were calculated as a fold-increase relative to GADPH gene expression. Threshold cycles (Ct) were determined for the genes studied, from the amplification curves. The amount of RNA in stimulated cells relative to control cells was calculated by the 2ΔCt method. We used the TCTTGGCAGCCTTCCTGATT and TTTCGTGTTGGCGCAGTGT primers for IL-8 and the GCCACATCGCTCAGACAC and GCCCAATACGACCAAATCC primers for GAPDH, which was used as a control.

### LC-MS/MS characterization of proteins of interest

Proteins were separated by electrophoresis in denaturing conditions (10% SDS-PAGE, gel 13 x 13 cm) and detected by Coomassie blue. Proteins from a second gel were transferred onto nitrocellulose membranes and analyzed by Far-western blotting, as described above. Proteins recognized by TLR2 were matched to stained bands, excised from the gel, reduced by incubation at 50°C for 1 h with 10 mM DTT and then alkylated by incubation for 1 h in the dark with 55 mM iodoacetamide. Fragments were washed several times with water and ammonium carbonate, dehydrated with acetonitrile (ACN) and dried. Trypsin digestion was performed overnight with a dedicated automated system (MultiPROBE II, Perkin Elmer). The gel fragments were then incubated twice, for 15 minutes each, in a H_2_O/CH_3_CN solution, to extract the peptides from the gel fragments. Peptide extracts were then dried and dissolved in the initial buffer for chromatographic elution, which consisted of 3% CH_3_CN and 0.1% HCOOH in water. Peptides were enriched, separated and analyzed with a 6520 Accurate-Mass Q-TOF LC/MS machine equipped with an HPLC-chip cube interface (Agilent Technologies, Massy, France). The fragmentation data were interpreted with Mass Hunter software (version B.03.01, Agilent Technologies). For protein identification, MS/MS peak lists were extracted, converted into mzdata.xml format files and compared with the protein database, using the MASCOT Daemon (version 2.1.3; Matrix Science, London, UK) search engine. The searches were performed with no fixed modification, with variable modifications for methionine oxidation, and with a maximum of one missed cleavage. MS/MS spectra were searched with a mass tolerance of 20 ppm for precursor ions and 0.6 Da for MS/MS fragments. Only peptides matching an individual ion score >56 were considered. Proteins with two or more unique peptides matching the protein sequence were automatically considered as a positive identification.

### N-terminal sequencing

For N-terminal determination, 20 μg of protein mixture was subjected to electrophoresis and the bands of interest were excised and incubated in a buffer for passive extraction of the protein from the acrylamide gel. After overnight incubation with shaking, the solution was eluted on a ProSorb Filter (Applied Biosystems) to fix the protein on a PVDF disc. The PVDF disc was submitted to microsequencing by Edman degradation (8 cycles of pulsed-liquid chemistry), with a 494 automated protein sequencer (Applied Biosystems).

### CAMP-1 PCR amplification and sequencing

For amplification of the nucleotide sequence of CAMP factor 1 from the 27 *P*. *acnes* strains, we performed PCR with the C1-F and C1-R primers, amplifying a 946 bp fragment, as previously described [35]. Briefly, standard amplification was carried out in a final volume of 25 μl containing 3 μl of extracted DNA. Each reaction mixture contained 2.5 μl of 10 X PCR buffer [500 mM KCl, 200 mM Tris-HCl (pH 8.4)], 1.5 μl of 50 mM MgCl_2_ (giving a final concentration of 3 mM), 0.5 μl of each primer (0.2 mM final concentration), 0.2 μl (1 U) of native *Taq* DNA polymerase (Invitrogen, Carlsbad, California, USA), and distilled water. The PCR conditions for amplification were as follows: 95°C for 3 minutes, followed by 35 cycles of 95°C for 1 minute, 60°C for 30 s, and 72°C for 1 minute and a final extension phase at 72°C for 10 minutes. The PCR products (5 μl) were analyzed by electrophoresis in a 2% agarose gel and the amplified nucleotide bands were visualized by UV transillumination of the ethidium bromide-stained gel, with comparison to a 50-bp DNA ladder (Invitrogen, Carlsbad, California, USA). Sequencing reactions were performed with ABI PRISM Ready Reaction Terminator cycle sequencing kits (Applied Biosystems) according to the manufacturer’s instructions. Sequencing reactions were performed with the Big Dye V3.1 Terminator Kit (Applied Biosystems, Foster City, CA, USA) according to the manufacturer’s instructions and analyzed on an ABI PRISM 3100 DNA sequencer (Applied Biosystems). DNA sequences were analyzed with SeqScape ® v2.5 Software from Applied Biosystems (Foster City, CA).

### Phylogenetic analysis

Phylogenetic analyses were performed to assess the relationships between the *P*. *acnes* CAMP factor 1 sequences obtained in this study and other known CAMP sequences. These analyses were carried out with the maximum likelihood method. Multiple sequence alignments were obtained with Mafft version 7 [61] and exported into Seaview software [62], for input into JModelTest [63] and ProtTest [64] to identify an appropriate substitution model for phylogenetic analysis with the Bayesian information criterion. The best model for nucleotides was HYK85 and the best model for proteins was WAG, both without a gamma distribution. The phylogenetic analysis was performed with PhyML3.0 [65], implemented in Seaview software [16]. Sequences were randomized by 500 bootstrapping resamplings, and statistical analyses were performed on 100 datasets for each analysis.

### Nucleotide sequence accession numbers

All the sequences determined in this study have been deposited in the GenBank database under the accession numbers listed in Table 4.

**Table 4:** GenBank accession numbers of CAMP factor 1 genes from all the *P*. *acnes* isolates studied

Isolate	GenBank accession no. for gene
PA 12513	KX581387
PA 14230	KX581388
PA 16351	KX581389
PA 17248	KX581390
PA 22197	KX581391
PA 22795	KX581392
PA 25236	KX581393
PA 27387	KX581394
PA 27647	KX581395
PA 38862	KX581396
PA 41103	KX581397
PA 47474	KX581398
PA 53468	KX581399
PA 75150	KX581400
PA 78910	KX581401
PA A9	KX581402
PA A11	KX581403
PA A 24	KX581404
PA A26	KX581405
PA A30	KX581406
PA A44	KX581407
PA CHR	KX581408
PA GUE	KX581409
PA PIE	KX581410
PA RON	KX581411
PA TRI	KX581412

### Statistical analysis

The statistical significance of differences between data from experimental groups was analyzed in paired Student’s *t*-tests. *P* < 0.05 was considered significant. Statistical significance is indicated by * (*P* ≤ 0.05), ** (*P* ≤ 0.01), *** (*P* ≤ 0.001) and **** (*P* ≤ 0.0001), respectively.

## Supporting Information

S1 Fig TLR2 expression in keratinocytes. The expression of TLR2 was assessed by western blotting, as described in the Materials and Methods. Lanes 2 and 4: 293T cell and ThP1 lysates used as positive controls. Lane 3: HaCaT keratinocyte lysate. Lane 1: MagicMark molecular mass markers. Arrow indicates position of TLR2.
(TIF)

S2 Fig TLR2 binding to *P*. *acnes* surface proteins. *P*. *acnes* surface proteins were extracted from a five-day culture bacterial pellet and separated by electrophoresis in 4–12% NuPAGE LDS BisTris gels (50 μg). The separated proteins were transferred onto nitrocellulose membranes, which were incubated with recombinant TLR2 (0.1 μg/ml). TLR binding activity was detected with specific biotinylated antibodies against TLR2, as described in the Materials and Methods. Lane 1 contains molecular mass markers. Lanes 2 to 22 contain proteins from strains 75150, 22197, 14230, 78910, 16351, 17248, 12513, 22795, 53468, 36862, 41103, 47474, 27647, 27387, 25236, A9, A11, A24, A26, A30, and A44, respectively. Arrows indicate the positions of the 24.5- and the 27.5-kDa bands of interest.
(TIF)

S3 Fig Phylotype determination of *P*. *acnes* strains. Five-day-old cultures from the 27 strains were used to extract DNA for multiplex-PCR, as described in the Materials and Methods, and the PCR products were analyzed by electrophoresis in a 1.5% agarose gel. Lane 1: 50-bp DNA ladder. Lanes 2 to 28: strains 12513, 14230, 16351, 17248, 22197, 22795, 25236, 27387, 27647, 38862, 41103, 47474, 53468, 75150, 78910, A9, A11, A24, A26, A30, A44, 6919, CHR, PIE, RON, TRI, and GUE, respectively. Letters a, b, c, and e correspond to amplicons of 677, 494, 145, and 351 bp, respectively, as previously described [36].
(TIF)
