# Supplementary figures and images for: TLR-2 Recognizes Propionibacterium acnes CAMP Factor 1 from Highly Inflammatory Strains
Source: PLoS One. 2016 Nov 30;11(11):e0167237. doi: 10.1371/journal.pone.0167237 (PMC5130237; doi:10.1371/journal.pone.0167237)

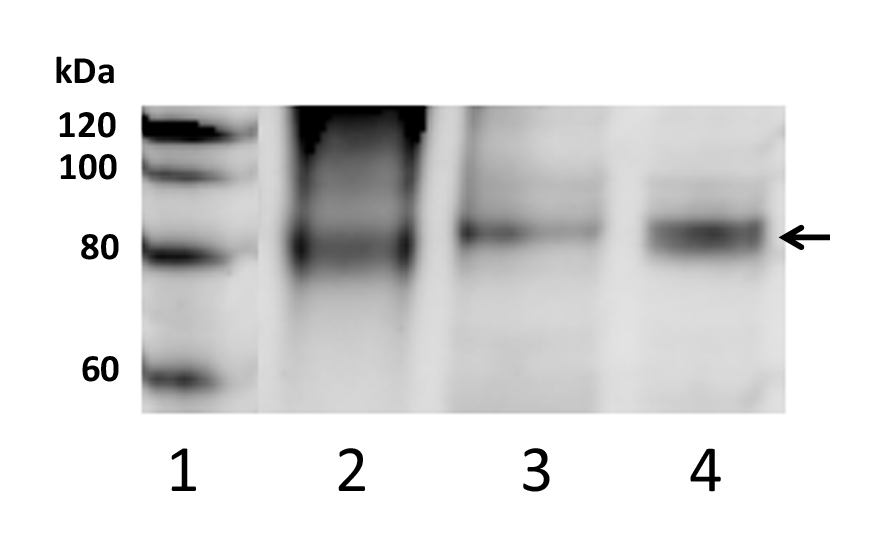

Supplement: S1 Fig — The expression of TLR2 was assessed by western blotting, as described in the Materials and Methods. Lanes 2 and 4: 293T cell and ThP1 lysates used as positive controls. Lane 3: HaCaT keratinocyte lysate. Lane 1: MagicMark molecular mass markers. Arrow indicates position of TLR2. (TIF) [file pone.0167237.s001.tif]

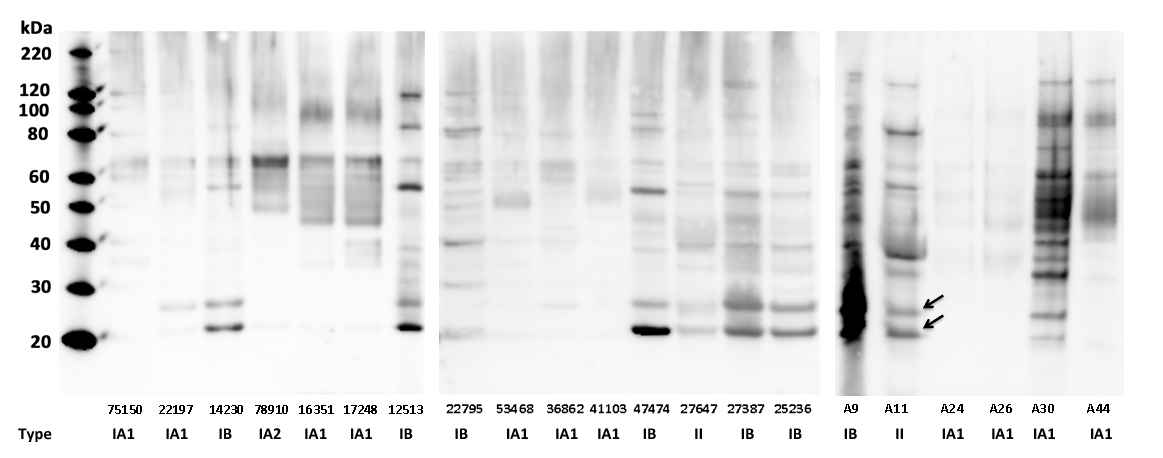

Supplement: S2 Fig — P. acnes surface proteins were extracted from a five-day culture bacterial pellet and separated by electrophoresis in 4–12% NuPAGE LDS BisTris gels (50 μg). The separated proteins were transferred onto nitrocellulose membranes, which were incubated with recombinant TLR2 (0.1 μg/ml). TLR binding activity was detected with specific biotinylated antibodies against TLR2, as described in the Materials and Methods. Lane 1 contains molecular mass markers. Lanes 2 to 22 contain proteins from strains 75150, 22197, 14230, 78910, 16351, 17248, 12513, 22795, 53468, 36862, 41103, 47474, 27647, 27387, 25236, A9, A11, A24, A26, A30, and A44, respectively. Arrows indicate the positions of the 24.5- and the 27.5-kDa bands of interest. (TIF) [file pone.0167237.s002.tif]

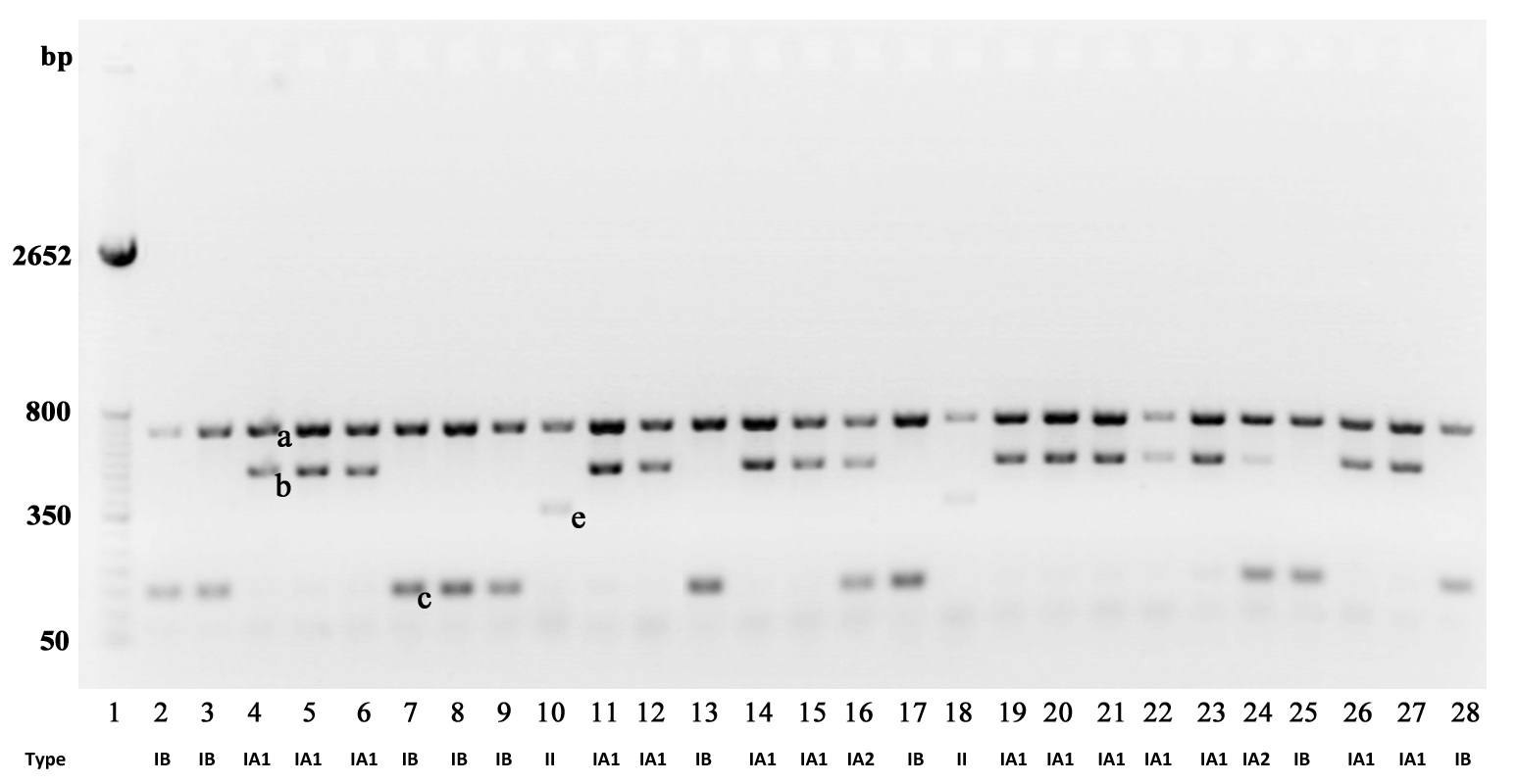

Supplement: S3 Fig — Five-day-old cultures from the 27 strains were used to extract DNA for multiplex-PCR, as described in the Materials and Methods, and the PCR products were analyzed by electrophoresis in a 1.5% agarose gel. Lane 1: 50-bp DNA ladder. Lanes 2 to 28: strains 12513, 14230, 16351, 17248, 22197, 22795, 25236, 27387, 27647, 38862, 41103, 47474, 53468, 75150, 78910, A9, A11, A24, A26, A30, A44, 6919, CHR, PIE, RON, TRI, and GUE, respectively. Letters a, b, c, and e correspond to amplicons of 677, 494, 145, and 351 bp, respectively, as previously described [36]. (TIF) [file pone.0167237.s003.tif]
